# Prophyll in Monocots: The Starting Point of Lateral Shoot Phyllotaxis

**DOI:** 10.3389/fpls.2022.855146

**Published:** 2022-04-13

**Authors:** Vladimir Choob

**Affiliations:** Botanical Garden, Lomonosov Moscow State University, Moscow, Russia

**Keywords:** prophyll, positional control, phyllotaxis, Commelinaceae, Amaryllidaceae, *Philodendron*

## Abstract

In monocots, the prophyll (or flower bracteole) is the first leaf of the lateral shoot. Typically, the prophyll occurs in an adaxial position toward the main axis; it bears two teeth at its apex and often two keels on the dorsal side. Some authors have hypothesized that the prophyll appeared in evolution as a result of the fusion of two phyllomes. However, in different monocot taxa, prophyll morphology results from the mechanical pressure of the surrounding organs and it cannot be regarded as two fused leaves. In Commelinaceae, if the lateral shoot develops extravaginally (i.e., penetrates the sheath) and the prophyll is not under pressure, the apical teeth and keels are missing. If the lateral shoot starts development intravaginally and under moderate pressure, the prophyll exhibits keels and a bidentate shape. In the bulbs of Amaryllidaceae, which are under strong pressure, the teeth of the prophyll become more pronounced, and the prophyll is dissected into two distinct lobes. In some monocots, the evolutionary trend leads to complete prophyll reduction. Investigations of lateral shoot phyllotaxis have found that the positions of all the subsequent phyllomes of the lateral shoot are sensitive to the prophyll position; they become rearranged if the prophyll deviates from the standard adaxial location (e.g., becoming oblique or transversal). As a generalization in Amaryllidaceae, I have proposed the axiomatic “phantom” method for modeling the prophyll position and shoot branching in cases of complete prophyll reduction. Using the phantom method, I reinvestigated the structure of sympodial units in *Philodendron* (Araceae). Previous interpretation of the two-keeled cataphyll as a prophyll appeared to be erroneous. In a new interpretation of the sympodial unit, the prophyll and the subsequent leaf are reduced and the cataphyll is the third leaf in the leaf series. A comparative morphological study in Araceae has revealed that prophylls of vegetative shoots rarely elongate and resemble round scales with obscure boundaries with the main axis. This observation could explain prophyll reduction in *Philodendron*. As such, the positional control of phyllotaxis by the prophyll may be revealed even when the prophyll is completely reduced.

## Introduction

According to the modern definition, the prophyll is the first one or two leaves of a lateral shoot. Traditionally, prophyll(s) are serial homologs of cotyledons. Typically, monocots possess a single prophyll in an adaxial position, whereas eudicots develop two transversal prophylls (Goebel, 1923; Arber, 1925; Blaser, 1944; Tomlinson, 1970; Choob, 2002).

Despite intensive study in the past, there are many unresolved problems concerning the prophyll in monocots. Theoretically, it has been postulated that the bidentate/two-keeled structure of the prophyll is caused by mechanical factors. The proposed model assumes the loss of the central vein in the prophyll as a result of pressure from the parent axis (Arber, 1925; Blaser, 1944). However, there have been no direct observations or experiments in favor of this hypothesis.

Many researchers have postulated a possible reduction of the prophyll or the bracteole (Eichler, 1875; Arber, 1925; Remizowa et al., 2013b). I follow Eichler’s (1875) terminology to distinguish two cases of reduction. *Abort* means that a certain organ is pre-patterned and initiated, but then arrested in development. The organ rudiment and/or the organ primordium may be observed by microscopy. *Ablast* means that a certain organ is pre-patterned but does not form any visible structure. Ablasted organs may be referred to as cryptic organs. Studies of ablasted organs are objectively difficult and usually meet criticism. Eichler (1875) introduced ablasted organs to his diagrams, based mainly on theoretical assumptions. Most recently, cryptic organs have been indicated by molecular genetic studies (Long and Barton, 2000). In order to distinguish theoretically introduced organs, I proposed the term *phantom* (Choob, 1999). After postulating phantom positions, further evidence from developmental and molecular studies will be essential.

The question remains whether the reduced prophyll (whether aborted or ablasted) retains its ability to influence the position of the subsequent leaves. A precise definition of prophyll function in monocots would help in investigations of shoot system branching, especially in cases of shortened internodes and/or reduction of phyllomes (Choob, 1999, 2001, 2010). The scope of this review is to analyze the interconnection between pressure and prophyll shape, the diversity of prophyll morphology, the role of prophylls in positional signaling, and then to demonstrate the applicability of the elaborated theoretical principles to practical research in selected monocot model families, Commelinaceae, Amaryllidaceae, and Araceae.

## Prophyll Concepts in Monocots

Historically, the problem of homologizing bracteoles, the first leaves of vegetative shoots and cotyledons, was raised by P.J.F. Turpin, who stressed the morphological differences between foliage (true) leaves on the one hand and cotyledons and prophylls on the other (Turpin, 1819), focusing on morphological differences in a narrow list of investigated taxa (mainly Gramineae and Cyperaceae).

Early works mixed two different aspects of prophyll morphology: (i) descriptive – a leaf which differs from the foliage ones in habitus, and (ii) positional – a leaf in a certain position. This ambiguity in terminology was criticized by Eichler (1875), who attributed two prophylls for dicots and a single one for monocots. Subsequent efforts of plant morphologists led to the separation of the descriptive term *cataphyll(s)* (Niederblatt) – the lowermost leaf (or leaves) of the shoot, lacking leaf blades, and some other characters of *foliage leaves* (Laubblatt). The uppermost specialized leaves, subtending flowers, were designated as *bracts* (Hochblatt) (Arber, 1925; Serebriakov, 1952; Troll, 1954). At the same time, the term *prophyll* obtained a strict interpretation as a homolog of cotyledons, starting the leaf series of the lateral shoots (Ruter, 1918; Goebel, 1923; Arber, 1925; Blaser, 1944). To emphasize this proposed homology, German botanists called the part of the lateral axis between the prophyll node and the parent shoot *hypopodium* (which corresponds to *hypocotyl* in the seedling axis), and the internode between the prophyll(s) and following leaf – *epipodium* (which corresponds to *epicotyl*) (Troll, 1954).

Prophylls can vary in their morphology (Tomlinson, 1970). Ruter (1918) proposed distinguishing prophylls on the following descriptive principles: (i) Niederblatt-Vorblatt or *cataprophyll* (lacking leaf lamina); (ii) Laubblatt-Vorblatt or *photoprophylls* (photosynthetic green leaves with lamina); (iii) Hochblatt-Vorblatt or *bracteole* (specialized leaves of inflorescence). In this review, a *bracteole* is a prophyll of a lateral flower. *Bracteoles* are preceded by *bracts* (the subtending leaves of flowers). These pair of terms appear to be relative: a leaf may be the bracteole for a flower and at the same time the bract for the flower of the next order.

The concept of homologization attributes a single prophyll to monocots and two prophylls to eudicots. However, there are several known exceptions to this rule (Eichler, 1875; Arber, 1925; Esau, 1965; Choob, 2010; Remizowa et al., 2013a; Sokoloff et al., 2015). Monocots with two prophylls include *Dioscorea sativa* L. (Dioscoreaceae), *Tricyrtis hirta* (Thunb.) Hook. (Liliaceae), *Aphelia cyperoides* R.Br. (Restionaceae), and *Centrolepis milleri* M. D. Barrett (Restionaceae). Eudicots with a single prophyll include *Cercis siliquastrum* L. (Fabaceae), *Rumex confertus* L. (Polygonaceae), and buttercups with entire leaves (Ranunculaceae: *Ranunculus lingua* L., *R. aquaticus* Neck., *R. auricomus* L., *R. flammula* L., *R. amplexicaulis* L.). The list of exceptions within eudicots indicates that the character «single prophyll» evolved in distantly related taxa of eudicots, probably resulting from independent factors. Arber (1925) suggested that the sheathing base of the single prophyll does not leave any available space for another leaf in the same node.

**FIGURE 1:**
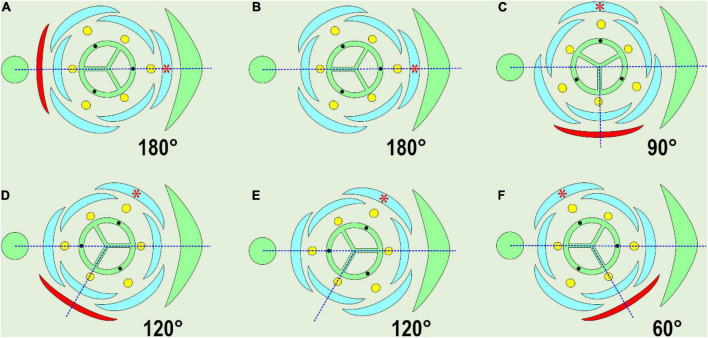
Variable position of the prophyll (bracteole), shown in red. The angle of divergence between the subtending leaf median and the prophyll is indicated. Note that in every case, one of the outer tepals opposes the prophyll (red asterisk). The majority of examples are compiled from Remizowa et al. (2006, 2013a), or from Eichler (1875) in **(E)**. **(A)** Adaxial (addorsed) position of floral prophyll: Iridaceae. **(B)** Floral prophyll absent, a tepal of the outer whorl in median abaxial position: *Veratrum* (Melanthiaceae), *Ornithogalum umbellatum* (Asparagaceae). **(C)** Tangential position, perpendicular to the median: *Japonolirion osense* (Petrosaviaceae), *Narthecium ossifragum* (Nartheciaceae), some Liliaceae (occasionally). **(D)** Tangential adaxial position, *J. ossense*, *N. ossifragum*, some Liliaceae (occasionally). **(E)** Floral prophyll absent, a tepal of the outer whorl in median abaxial position: *Heliconia metallica* (Heliconiaceae). **(F)** Tangential abaxial position: *J. osense*, *N. ossifragum*, some Liliaceae (occasionally).

It is important to mention that the main axis lacks a subtending leaf, so the positional criterion of homology in pairs «hypocotyl – hypopodium» and «cotyledon – prophyll» is incomplete. Nevertheless, both cotyledons and prophylls play a guiding role in phyllotaxis. If the position of the prophyll is changed relative to the subtending leaf, all the subsequent phyllomes of the lateral shoot correlatively change their angle coordinates (Choob, 2002, 2010). As examples of such a correlation in monocots, one can compare the relative positions of the parent axis, the bract (the subtending leaf), the bracteole (the prophyll of the flower), and the tepals (Figure 1). The angle between the subtending leaf median and the bracteole varies between 60° (deviating) and 180° (typical adaxial prophyll position) and in some cases the bracteole occupies a transversal position (90°), whereas one of the outer tepals is always positioned at an angle of 180°, relative to the bracteole (red asterisk in Figure 1). All the other flower organs rotate together with the bracteole.

In this review, we distinguish the following prophyll positions (Figure 1): adaxial (addorsed, 180°), transversal (at the angle of 90° to the subtending leaf median), median (0°), and tangential (all the other angles of divergence). The variation in the divergence angles of bracteoles was accurately documented in *Hedychium* by Kirchoff (2000), who applied Hofmeister’s rule to cases of prophyll position in Zingiberaceae, proposing that new primordia should appear as far as possible from ones already initiated. According to his observations, the prophyll of lateral flowers appears in an almost transversal position, as far as possible from both the subtending leaf and the parent axis, as dictated by Hofmeister’s rule. However, Kirchoff’s view does not cover all the variability of prophyll positions in monocots, including the positions shown here (Figures 1A,F).

Besides the unique position in leaf series, the monocot prophyll often has some specific morphological characteristics: a bidentate apex (sometimes with a large incision in between), two keels on the dorsal side, two «main» veins. Taken all together, these features have allowed some authors to hypothesize that the monocotyledonous prophyll evolved from two transversal prophylls by fusion (Ruter, 1918; Goebel, 1923). This hypothesis was challenged by many botanists and is now widely considered to be invalid (Arber, 1925; Tomlinson, 1970; Choob, 2002, 2010), as there should be two axillary buds in the prophyll axil if monocot prophylls were equivalent to two leaves. The only observation of two shoots in a prophyll axil was made in grasses by Ruter (1918), but it was not confirmed by further studies.

Other convincing evidence against the «two-phyllome» origin of the prophyll in monocots arises from studies of monocot prophyll development. Usually, the prophyll initiates as a single phyllome (Gravis, 1898; Arber, 1925), but in some cases in *Zea mays* L., it may appear as two separate, non-fused parts, even in late development (Johnston et al., 2010). In some Amaryllidaceae (see below) the prophyll exists as two separate lobes that are not connected; a common primordium of these two prophyll lobes could not be observed using microscopy.

A potentially parallel morphological series occurs in an early-divergent eudicot, *Ranunculus* (Choob, 2002, 2010). *Ranunculus* species with entire leaves have the addorsed prophyll in the basal lateral shoots, but at the inflorescence, they shift to two transversal prophylls due to gradual divergence change and epipodium shortening.

## Pressure and Prophyll Morphology in Commelinaceae

Pressure depends first on the direction of the lateral shoot growth. In monocots with sheathing leaves, there are two possible scenarios of lateral shoot growth. If the axillary apex grows inside and never disturbs the sheath of the subtending leaf, it is referred to as *intravaginal*. If the lateral apex breaks through the sheath outside, the shoot may be termed *extravaginal*. Intravaginal shoots are characterized by orthotropic (aerial) growth as a rule, while extravaginal shoots tend to grow horizontally, which often correlates with subterranean growth (Serebriakova, 1969; Peretta et al., 2011; Ott et al., 2019).

**FIGURE 2:**
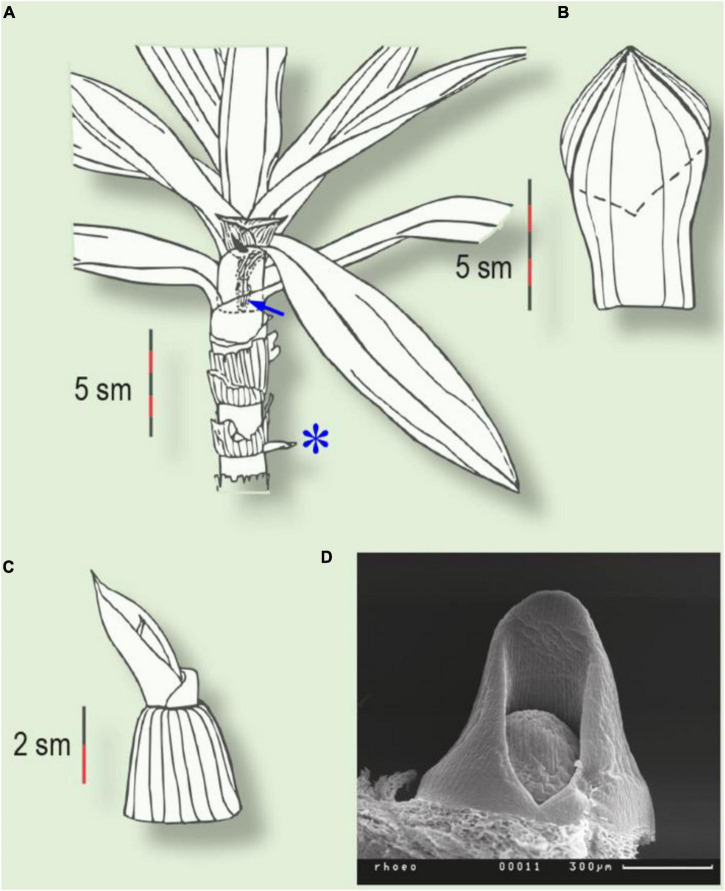
Two types of prophyll in *Tradescantia spathacea*. **(A)** Plant with intravaginal generative and extravaginal vegetative shoots (asterisk). Arrow indicates the prophyll of the generative shoot, inside the subtending leaf sheath. **(B)** Two-keeled prophyll of the intravaginal shoot. Dotted line indicates the ventral margin of the prophyll. **(C)** Prophyll of the extravaginal shoot. **(D)** Prophyll primordium of the vegetative shoot (SEM).

**FIGURE 3:**
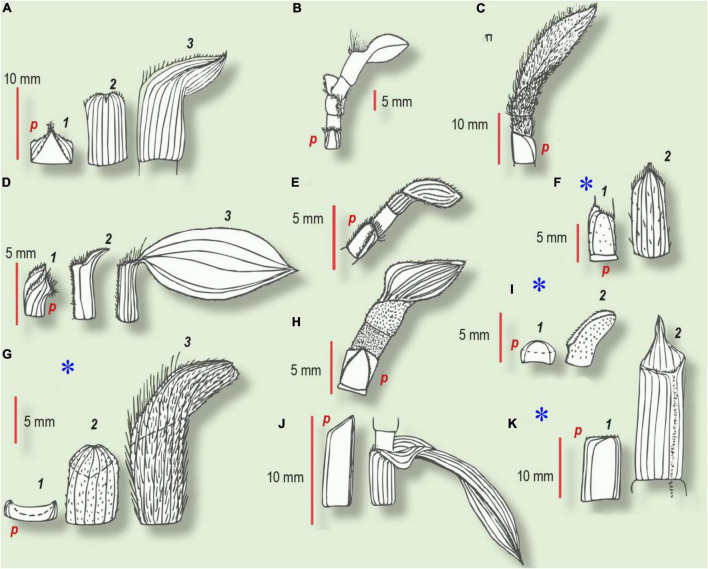
Habitus of prophyll and several subsequent leaves in Commelinaceae (from Choob, 2010). Numbers refer to the first, second, and third leaves in the series. **(A–E,H,J)** Intravaginal shoots. **(F,G,I,K)** Extravaginal shoots (marked with an asterisk). **(A)** *Tradescantia crassula*. (1) Prophyll with two keels, bearing trichomes; (2) second cataphyll, midvein glabrous; (3) intermediate leaf with a small lamina. **(B)** *T. albiflora*. cataprophyll; intermediate leaf with ventral barbule of cilia; foliage leaf. **(C)** *T. sillamontana*. At the base – the glabrous prophyll with oblique margin, hypopodium has no trichomes, whereas epipodium and all the subsequent green leaves have tomentose indumentum. **(D)** *T. fluminensis*. (1) Prophyll with two barbules along the keels and with cilia at the ventral joint; (2) second cataphyll with a dense indumentum of the ventral joint; (3) foliage leaf. **(E)** *Callisia repens*. At the base – two-keeled prophyll with two rows of trichomes, hypopodium glabrous; upper foliage leaf has ventral barbule of trichomes, continued through epipodium toward one of the prophyll keels. **(F)** *T. zebrina.* (1) prophyll; (2) second leaf of the lateral shoot with sparse trichomes. **(G)** *T. pallida*. (1) Glabrous prophyll with smooth margins; (2) second cataphyll with short trichomes; (3) foliage leaf with dense long indumentum. **(H)** *Callisia elegans*. The basal prophyll bears two keels with trichomes, hypopodium is glabrous; epipodium and foliage leaf with velutinous indumentum. **(I)** *T. navicularis*. (1) Prophyll without trichomes or mucrons; (2) foliage leaf with whitish mucrones and short cilia on the margin and short trichomes along the ventral joint. **(J)** *T*. × *andersoniana.* (1) Cataprophyll with oblique margin; (2) foliage leaf. **(K)** *C. fragrans*. (1) Prophyll with smooth margin; (2) second cataphyll with acute apex and indumentum along the ventral joint; *p*, prophyll.

**FIGURE 4:**
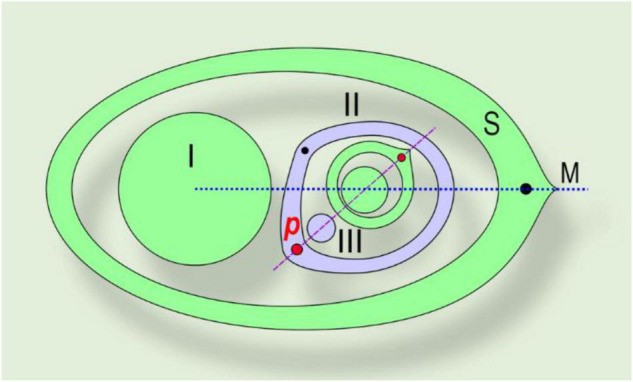
Asymmetric prophyll in Commelinaceae. The phyllotaxis of the lateral shoot is distichous, in a tangential position. A right-handed example is shown. (I) Main axis; (II) lateral shoot (axis of second-order); S, subtending leaf; M, median of subtending leaf, *p*, prophyll; (III) bud in axil of prophyll. Red dot – major vein of the prophyll, which determines the phyllotaxis of the subsequent leaves (also shown by red dot). Black dot – minor vein of the prophyll.

Differences in prophyll structure in Commelinaceae are summarized in Table 1 and Figures 2–4 (Choob and Mavrodiev, 2001). In some species, the lateral tillers develop in an intravaginal manner only, whereas in others, the shoots commence growth intravaginally, but at later stages, break through the subtending sheath. In *T. fluminensis*, sheath rupture occurs irregularly and is also rather late. Intriguingly, in *Tradescantia crassula*, after a very short period of intravaginal growth, the lateral shoot pushes out of the sheath due to the strong curvature of the hypopodium so that it is difficult to observe the primary direction of growth in this species.

**TABLE 1:** Prophyll morphology in Commelinaceae.

Species	Shoot type	Keels	Apex shape	Prophyll indumentum	Subsequent leaf indumentum
*Callisia elegans* Alexand. ex H.E. Moore	Intravaginal, late sheath break	Present	Acute	Trichomes along keels, marginal cilia	Velutinous dense trichomes
*Callisia fragrans fragrans* (Lindl.) Woodson	Extravaginal	Absent	Smooth, symmetric	Short marginal trichomes	Marginal and ventral row of trichomes
*Callisia repens* L.	Intravaginal	Present	Obtuse	Trichomes along keels, marginal cilia	Marginal and ventral row of cilia
*Cyanotis somaliensis* C. B. Clarke	Intravaginal	Present	Oblique, asymmetric	Absent	Marginal cilia
*Dichorisandra reginae* (Lind. et Rodig.) H. E. Moore	Extravaginal	Absent	Smooth, symmetric	Absent	Marginal cilia at the base of the lamina
*Tradescantia albiflora* Kunth	Intravaginal, late sheath break	Present	Acute	Trichomes along keels, marginal cilia	Marginal and ventral row of cilia
*Tradescantia* × *andersoniana* Ludw. et Rohw	Intravaginal	Present	Oblique, asymmetric	Absent	Marginal cilia at the base of the lamina
	Extravaginal	Absent	Smooth, symmetric	Absent	Marginal cilia at the base of the lamina
*Tradescantia crassula* Link	Intravaginal	Present	Acute	Trichomes along keels	Marginal cilia
*Tradescantia fluminensis* Vell	Intravaginal, occasional late sheath break	Present	Acute	Trichomes along keels, marginal and ventral cilia	Marginal and ventral row of cilia
*Tradescantia navicularis* Ortg.	Extravaginal	Absent	Smooth, symmetric	Absent	Marginal and ventral row of short trichomes, diffuse mucrons
*Tradescantia pallida* (Rose) D. R. Hunt	Intravaginal, late sheath break	Absent	Smooth, symmetric	Absent	Dense tomentose trichomes
*Tradescantia sillamontana* Matuda	Intravaginal	Present	Oblique, asymmetric	Absent	Dense tomentose trichomes
	Extravaginal	Absent	Smooth, symmetric	Absent	Dense tomentose trichomes
*Tradescantia spathacea* Sw.	Intravaginal	Present	Acute	Absent	Absent
	Extravaginal	Absent	Straight, symmetric	Absent	Absent
Tradescantia virginiana L.	Intravaginal	Present	Oblique, asymmetric	Absent	Marginal cilia at the base of the lamina
	Extravaginal	Absent	Smooth, symmetric	Absent	Marginal cilia at the base of the lamina
*Tradescantia zebrina* Heynh. ex Bosse	Intravaginal	Present	Oblique, asymmetric	Sparse diffuse trichomes, marginal cilia	Sparse diffuse trichomes, marginal cilia

Several taxa display exceptional extravaginal growth. In some species, both types of lateral shoot develop within the same plant: extravaginal shoots are attached to the basal part of the parent stem, whilst intravaginal ones occupy the apical part. Thus, in *T. spathacea*, all the vegetative shoots are extravaginal, but all the inflorescence shoots are intravaginal (Figure 2). The transition of the lateral bud outgrowth from an intravaginal to an extravaginal mode in some Poaceae can be caused by regular clipping and is accompanied by more prostrate lateral growth (N’Guessan and Hartnett, 2011).

According to our observations (Choob and Mavrodiev, 2001), in all the intravaginal shoots of Commelinaceae, irrespective of secondary rupture of the sheath, the prophyll develops two more or less conspicuous keels. In *Tradescantia crassula*, *T. fluminensis*, *Callisia elegans*, and *C. repens*, the prophyll bears trichomes either on both keels or even on two distinct barbules of trichomes converging to the apex (Figure 3). In contrast, in the case of the extravaginal tillers, in all the taxa investigated the prophylls lack pronounced keels and were usually glabrous or with a few sparse trichomes.

In species with both intra- and extravaginal shoots, prophyll shape is correlated with growth type: two keels appear on the prophylls of the intravaginal tillers only, while the extravaginal tiller prophylls lack distinct keels (Table 1). All the data obtained on the relationship between prophyll shape and direction of shoot growth in Commelinaceae are in accordance with the observations in other monocots (Blaser, 1944).

It is worth commenting on the intravaginal prophylls with an oblique apex in *T. sillamontana*, *T. virginiana*, and *T.* × *andersoniana*. The prophyll appears asymmetric because one of the two veins along the keels is stronger and longer than the other. The bud in the axil of the prophyll opposes the strongest vein (Figure 4). The asymmetric prophylls and the position of their axillary buds were initially described by Gravis (1898). Thus, an intriguing question in Commelinaceae arises regarding the position of the prophyll median, either (i) between two keel veins – corresponding to a strictly adaxial position, (ii) through one of the two veins, accompanied by axillary bud – corresponding to a tangential prophyll position, (iii) halfway between the right and left margins of the leaf, or (iv) where the primordial leaf apex was at the stage of initiation. In Commelinaceae, the prophyll is a leaf with an enclosed sheath, so the (iii) variant does not fit. The divergence angle between the prophyll axillary bud and the subsequent leaf on the lateral branch is almost 180°; correspondingly, the spatial organization of the lateral shoot phyllotaxis is guided by the position of the prophyll axillary bud, but not by the point between two keels (Figure 4). We confirmed this regularity in all the investigated Commelinaceae, including those with a symmetric prophyll shape (Choob and Mavrodiev, 2001; Choob, 2010).

Because of the distichous phyllotaxis, right-handed and left-handed asymmetric prophylls regularly follow each other on the stem in these *Tradescantia* species (Choob, 2010). The position of the prophyll axillary bud allows us to put the prophyll median tangentially relative to the median of subtending leaf of the parent axis (Choob and Mavrodiev, 2001). Thus, in this case, the midvein is not reduced, as proposed by Arber (1925), but shifted to a tangential position.

To address the question about the influence of pressure on keel development, we experimentally restricted the growth of the extravaginal tiller in *C. fragrans* and *T. zebrina*. Prior to bud outgrowth, we mounted adhesive tape around the subtending sheath and fixed it with a thread. The lateral shoot was forced to grow intravaginally, facing artificial pressure. Three weeks later, we removed the tape and investigated the prophyll shape. On the dorsal side, we observed two conspicuous keels with a shallow incision between them (bidentate structure). As a result, the shape change was achieved by direct experiment, supporting the hypothesis of pressure involved in bidentate structure development (Choob, 2010).

As also noted by De Craene (2018), organs often show pressure marks, reflecting their development in a confined space and economic use of this space in the bud. He emphasized that the precise stage in development when the pressure occurs is important for organology and organ shape. In the case of Commelinaceae, pressure influences late development only, so the organ positions remain unchanged, but organs often show pressure marks, reflecting their development in a confined space and economic use of this space in the bud.

In summary, in Commelinaceae, we have constructed a morphological series starting from distinctly keeled prophylls (*Tradescantia crassula*, *T. fluminensis*, *C. repens*, *C. elegans*), *via* oblique asymmetric weak-keeled prophylls (*Tradescantia sillamontana*, *T.* × *andersoniana*, *T*. *virginiana*) to prophylls lacking keels entirely (*T. navicularis*, *C. fragrans*) (Figure 3). In all these cases the prophylls occur in a tangential (but not in adaxial/addorsed) position (Figure 4).

## The Prophyll in Amaryllidaceae: Modes of Reduction

Amaryllidaceae are geophytes with terminal inflorescence and hence with sympodial bulb/rhizome innovation during the generative phase of development. In Amaryllidaceae, the prophyll morphology and position are well-documented in multiple research works (Irmisch, 1850, 1860; Blaauw, 1931; Artyushenko and Shchepak, 1982; Paula, 2006; Choob, 2010, 2020). The prophylls vary in shape from a normal foliage leaf (photoprophyll) to a more or less reduced scale (cataprophyll) or even complete abortion. Thus, Amaryllidaceae can serve as a model family for the investigation of the modes of prophyll reduction.

In Amaryllidaceae, prophylls occur in four principal positions: (i) at the base of flowers, leading to inflorescences of one to several helicoid cymes (Stout, 1944); (ii) at the base of lateral inflorescence stalk (paracladium); (iii) at the base of the main innovation bud, accompanied by a subsequent main inflorescence; (iv) at the base of other lateral shoots. The latter case (iv) is characterized by a uniform addorsed position of the photo- or cataprophyll, which never undergoes any substantial reduction. On the one hand, photoprophylls are formed in taxa where cataphylls are not characteristic (e.g., *Hippeastrum*, *Zephyranthes*). On the other hand, photoprophylls may develop in taxa with cataphylls if the lateral bud is formed in the same season of growth as the parent shoot without any dormancy. If the lateral bud has a period of resting, it usually produces a cataprophyll. We documented this phenomenon for *Narcissus* (Choob and Kozhevnikova, 2000; Choob, 2010).

**FIGURE 5:**
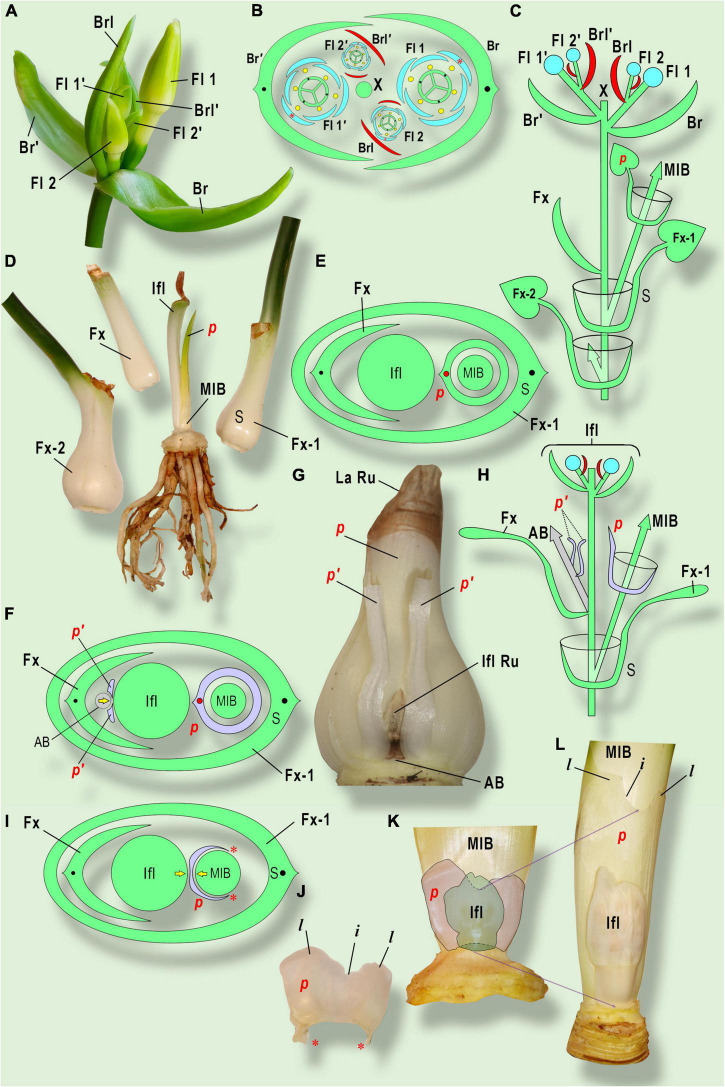
Prophyll morphology and position in Amaryllidaceae. **(A–E)** *Eucharis grandiflora* Planch. et Lindl.; **(F–H)** *Cyrtanthus elatus* (Jacq.) Traub; **(I–L)** *Clivia gardenii* Hook. **(A)** General view of inflorescence. Two sequential cymes are shown. The upper cyme is marked with «’» sign. **(B)** Inflorescence diagram. **(C)** Longitudinal scheme of inflorescence and sympodial growth of *Eucharis grandiflora*. **(D)** Three consequent leaves and stem plate with inflorescence stalk (after flowering) and photoprophyll (*p*) (Laubblatt-Vorblatt) in adaxial position. **(E)** Bulb diagram in *Cyrtanthus elatus*. The split prophyll (*p’*) belongs to the aborted bud (AB) or paracladium on the front side from inflorescence. Both prophylls are in an addorsed position to the inflorescence. The yellow arrow indicates the pressure of an aborted bud, resulting in prophyll splitting. **(G)** General view of major innovation bud and neighbor organs (the leaves of the main shoot are removed). The prophyll of the major innovation bud (*p*) is very similar to foliage leaves, besides early lamina reduction. **(H)** Longitudinal scheme of shoot branching. **(I)** Bulb diagram in *Clivia gardenii*. Note the pressure between the inflorescence and the major innovation bud, resulting in prophyll two-lobed shape. Red asterisks mark the non-fused prophyll margins. **(J)** The membranaceous prophyll (*p*) (caraprophyll, or Niederblatt-Vorblatt) of the major innovation bud has two lobes (*l*) with incision (*i*) in between. **(K)** The inflorescence and the major innovation bud at an early stage of development. Note the prophyll incision placed exactly under the inflorescence. **(L)** Late stage of development. The lobes with incision shift up due to basal growth of the prophyll, whereas the inflorescence growth is retarded. AB, aborted bud; Br, bract; Brl, bracteole (prophyll) of flower 1 and the subtending leaf of flower 2; Fl 1, the flower in the bract axil; Fl 2, the flower of the next order in the axil of bracteole; Fx, the leaf, preceding bracts (in Amaryllidaceae it has an unclosed base); Fx-1, Fx-2, sheathing leaves, preceding Fx; IFL, inflorescence; IFL Ru, inflorescence rudiment; La Ru, leaf lamina rudiment; MIB, major innovation bud; *p*, prophylls; S, subtending leaf of MIB (Fx-1); X, the position of the generative shoot apex.

In the case (i) of bracteoles (Hochblatt-Vorblatt *sensu*, Ruter, 1918), they occur as small slightly asymmetric scales and occupy a tangential position relative to the parent axis (flower or inflorescence). Usually, the bracteoles develop freely in multiflowered inflorescences, as in the African genera *Haemanthus*, *Scadioxus*, *Clivia*, and *Nerine*. Even in pauciflowered or uniflowered taxa, such as the American genera *Eucharis*, *Calliphuria*, *Hippeastrum*, *Zephyranthes*, *Traubia*, *Eremolirion*, *Rhodophiala*, and *Phycella*, lanceolate, linear or filiform bracteoles were described (Meerow, 1989; Arroyo-Leuenberger and Leuenberger, 2009; García et al., 2019; Figures 5A–C). Similar positions of bracteoles (which are the subtending bracts for the subsequent flowers in a cyme) were described in Zingiberaceae (Kirchoff, 2000). Despite the lateral position of the flower(s), in *Narcissus* and *Galanthus*, bracteoles rarely develop (Luiten and van Waveren, 1952; Müller-Doblies, 1971; Noy-Porat et al., 2009; Choob, 2010).

The formation of paracladia is a comparatively rare event in Amaryllidaceae. In case (ii), new inflorescences may appear in the axil of the semi-sheathing leaf, preceding the bracts. A single axillary paracladium sometimes occurs in *Galanthus*, some cultivars of *Narcissus* (regularly in the ‘Tête-à-tête’ cultivar). Usually, these paracladia lack visible prophylls, but from time to time an asymmetric scale can be observed at the base of the axillary inflorescence (Choob and Kozhevnikova, 2000; Choob, 2010). In cultivated *Leucojum aestivum* L. several paracladia are arranged in helicoid cyme-like structures are accompanied by narrow scale-like prophylls in a tangential position to the parent inflorescence (Müller-Doblies, 1971).

In the leaf axil preceding the inflorescence in *Haemanthus albiflos* Jacq. and *Cyrtanthus elatus* (Jacq.) Traub, there is usually a leaf that is split into two lobes, which occupies an addorsed position relative to the inflorescence stalk. We assume this structure represents the prophyll of an aborted paracladium or aborted axillary bud. The mechanical constraints of prophyll development lead to its deep symmetric splitting (Choob, 2010, 2020).

The most complex case is (iii) prophyll development at the base of the main innovation bud. The morphological characteristics of this prophyll are strictly correlated with the geographical clades proposed by Meerow and Snijman (2006), and undoubtedly, indicate an important synapomorphy, which may be useful in Amaryllidaceae systematics. The American clade includes the tribes Hippeastreae, Eucharideae, and Hymenocallideae, which always produce the prophyll with a leaf blade. In general, the most common representatives of the American clade do not develop cataphylls. Consequently, photoprophylls of the main innovation buds located exactly in the adaxial position have been documented in the American genera *Hippeastrum* (Irmisch, 1850, 1860; Blaauw, 1931; Artyushenko and Shchepak, 1982), *Zephyranthes* (Dzidziguri, 1979; Paula, 2006), and *Ismene* (Choob, 2020). In all the cases listed, the prophyll has a sheathing base and long linear lamina. In the tribe Eucharideae, leaves are differentiated into a circular sheath, petiole, and lamina (Meerow, 1989). Unfortunately, the prophyll structure in Eucharideae has not yet received sufficient attention, though we have established that the prophyll of the main innovation bud is differentiated into a petiole and lamina, revoluted toward the inflorescence stalk (Figures 5D,E; Choob, 2020).

The African tribes of Haemantheae and Cyrtantheae (like American Amaryllidaceae) develop the prophyll of the innovation bud in an adaxial position, but the shape of this leaf has undergone reduction. A sheathing bladeless fleshy scale in the bulb of *Cyrtanthus elatus* (Jacq.) Traub was described by Asatryan (1993), but its particular position remained unclear. Slabbert (1997) observed the leaves with lamina only. According to our observations, in *C. elatus* the fleshy leaf with a circular base and rudimentary lamina correspond to the prophyll of the main innovation bud (Figures 5F–H) (Choob, 2010, 2020). Our difference with Slabbert’s data could be explained by differences in climatic conditions and/or clonal variability. In any case, *C. elatus* gives an example of a transition from photoprophylls to cataprophylls.

In *Clivia* (Haemantheae), the specifics of prophyll development were investigated by Irmisch (1860), who described the membranaceous circular bidentate scale at the base of the main innovation bud in *C. nobilis* Lindl. This scale is significantly smaller than the subsequent foliage leaves. The teeth are oriented to the adaxial side (toward the inflorescence stalk). In *C. miniata* (Lindl.) Bosse, the membranaceous prophyll has an open base, but sheaths the main innovation bud from a quarter to a third of its circumference (Asatryan, 1993; Choob, 2020). The prophyll has two major acute teeth on the dorsal side, but sometimes it develops additional teeth, probably due to mechanical obstacles of development. In *C. gardenii* Hook., the prophyll is smaller than in other *Clivia* species, sheathing a quarter or less in circumference and with two obtuse lobes and a small incision in between (Choob, 2020). Obviously, these are cases of cataprophylls, the only cataphylls found in *Clivia* (Figures 5I–L).

Studies of the structure of bulb scales in *Haemanthus albiflos* have revealed that the prophyll of the main innovation bud is fleshy (young) or membranaceous, with two obovate lobes and deep incision caused by pressure from the inflorescence (Irmisch, 1860; Asatryan, 1993; Afanasjeva, 1995). Additionally, we observed the reduction of one of two lobes (half of the prophyll was reduced) or even the rudiment as a narrow thin membrane without lobes (Choob, 2010, 2020). All these variants of prophyll shape can occur on the same plant, which means that the degree of the prophyll development depends mostly on the growing conditions.

It is worth mentioning that *H. albiflos* and *C. elatus* produce the additional prophyll in the axil of the leaf, preceding the inflorescence. This prophyll consists of two separate lobes (obtuse or acute) and may be attributed to the aborted bud or paracladium (see above) (Figures 5G,H).

Resuming the characters of the prophyll of the main innovation bud in African clades of Amaryllidaceae, we emphasize that in most cases it is represented by a bladeless scale inside the bulb. A morphological reduction series of reduction starts from *C. elatus* (enclosed fleshy scale with aborted lamina), then species of *Clivia* (from enclosed to open-ranked membranaceous scale) to *Haemanthus* (reduced to bilobed or even rudimentary scale). However, we could not describe this trend as an evolutionary one because molecular data demonstrate that all the listed taxa belong in several parallel clades (Meerow and Clayton, 2004; Meerow and Snijman, 2006). Thus we postulate great diversity in prophyll structure among African Amaryllidaceae.

One of the basal tribes, Amaryllideae, includes the African genera *Amaryllis* and *Nerine* and the pantropical genus *Crinum*. In *Nerine bowdenii* W. Watson, the prophyll of the main innovation bud is presented by a small membranaceous scale, named «quarter leaf» by Theron and Jacobs (1994) because of sheathing to a quarter circumference. All the species studied by Irmisch (1850, 1860) possess a characteristic scale in an addorsed position with a narrow base and weakly expressed bidentate structure or even lacking teeth. In *Crinum*, the prophyll of the main innovation bud is also narrow, sometimes linear, with varying strength of keels and teeth (Irmisch, 1850, 1860; Artyushenko and Shchepak, 1982; Choob, 2020). In *Amaryllis*, the main innovation bud has the first foliage leaf, opposite the inflorescence. This raises the question of whether this leaf is the prophyll in an inadequate position or the main innovation bud is terminal. Investigation of the inflorescence development left no doubt about the axillary nature of the main innovation bud but gave no appropriate interpretation of the first leaf position (Hartsema and Leupen, 1942). In my opinion, the foliage leaf of the main innovation bud is the second phyllome in the leaf series. The prophyll in *Amaryllis* is completely reduced in the adaxial position but still gives a positional signal to all the subsequent leaves in the innovation bud (Choob, 2020).

In the Eurasian clade of Amaryllidaceae, the tribe Lycorideae is the first-divergent lineage (Meerow and Snijman, 2006). Japanese morphologists described the bulbs in six *Lycoris* species, where the main innovation bud started the leaf series with a membranaceous scale a third- to half-sheathing the bud (Mori and Sakanishi, 1976). The mid-Asiatic *Ungernia tadschicorum* Vved. ex Artjush. was thoroughly examined by Müller-Doblies and Müller-Doblies (1978). The prophyll of the main innovation bud developed as two distinct acute scales on both sides of the inflorescence stalk. This shape appeared as a result of deep mechanical dissection of a single prophyll. *Pancratium* may be referred to as an intermediate morphological state of the prophyll between Lycoridae and Narcisseae. In *P. maritimum* L., the prophyll is addorsed, bidentate, two-keeled, and comparatively narrow (Irmisch, 1860; Artyushenko, 1970; Artyushenko and Shchepak, 1982).

In the Mediterranean clade Narcisseae/Galantheae, the first cataphyll (or foliage leaf) of the main innovation bud is opposed to the inflorescence, so it is not addorsed. This character was first discovered in *Galanthus* (Saint-Hilaire, 1841), then confirmed for *Narcissus*, *Sternbergia*, *Leucojum*, and *Acis* (Irmisch, 1850, 1860; Luiten and van Waveren, 1938, 1952; Rees, 1969; Artyushenko, 1970; Vogel and Müller-Doblies, 1975; Mori et al., 1990). As this arrangement of leaves and shoot branching points resembles that of *Amaryllis*, we proposed the same interpretation: the prophyll is completely reduced – aborted or ablasted *sensu* Eichler (1875). Despite the prophyll reduction, the position of the subsequent leaf remains unchanged, leading us to the conclusion that the *positional signaling* from the prophyll was not disturbed. We cannot directly observe the initiation of the prophyll, but it still fulfills its positional function as a coordinate origin for the phyllotaxis of the [axillary] main innovation bud (Choob, 1999, 2010; Choob and Kozhevnikova, 1999, 2000).

This hypothesis is testable: we expect to find a rudimentary leaf in the adaxial position to the main inflorescence as a rare abnormality. In this context, it is interesting to note the observation of *N. campernelli* hort. ex Haw. with the regular formation of two separate scales between the innovation bud and inflorescence exactly in an adaxial position (Irmisch, 1860), which we also confirm in cultivar “Double Campernelli” (Choob, 2010, 2020). In addition, we twice observed a rudimentary addorsed scale in the *Galanthus* main innovation bud (Choob, 2010).

Summarizing the data for Amaryllidaceae, the relative position of the inflorescence and the main innovation bud is very conservative. The only trend is the reduction of the prophyll. The initial state is plesiomorphic, judging from the basal and African clades. The complete reduction of the main innovation bud prophyll is a synapomorphy for the Narcisseae/Galantheae clade. At the same time, *Pancratium* seems to be distinguished by the presence of a prophyll. The American clade has the prophyll as a foliage leaf (Choob, 2020).

## The Sympodium in *Philodendron* as a Formal Morphological Problem

The rules in prophyll position in monocots have led us to investigate phyllotaxis and branching points, a study that we have designated “phantom analysis,” and applied to Amaryllidaceae, Iridaceae, and Araceae (Choob, 1999, 2010; Choob and Kozhevnikova, 1999; Choob and Kuznetsova, 1999). This method is based on axiomatic postulates, followed by formal modeling. If necessary, the analyzed leaf series may be supplemented by phantom leaves, which are assumed to be virtual organs within the model until any rudimentary or additional leaf can be observed in the phantom position. The term «phantom» is necessary to separate theoretical conclusions from real observations.

**FIGURE 6:**
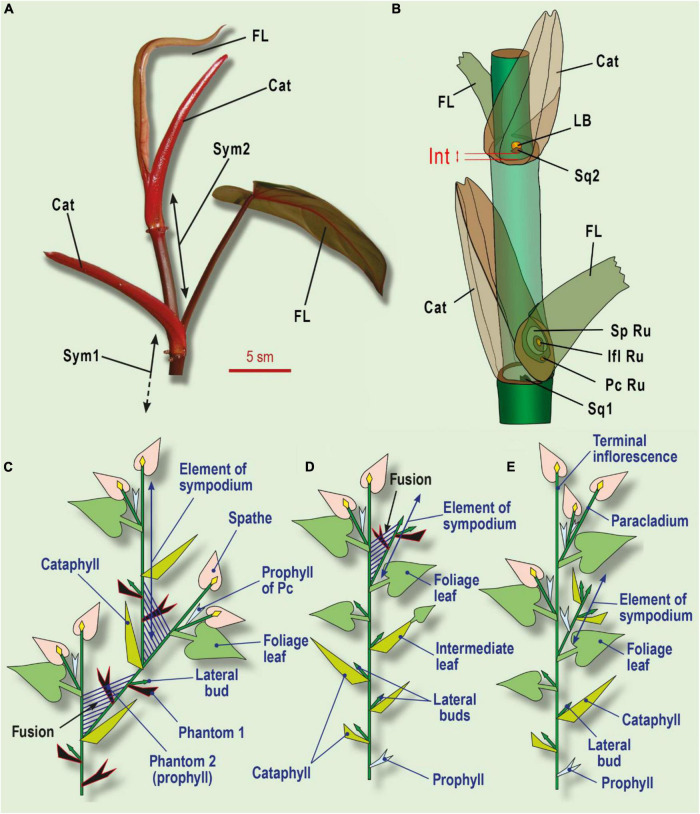
Leaf series of two subsequent sympodial elements in *Philodendron* (Araceae) and its interpretation. **(A)** General view of two symposium elements in *Ph. erubescens* C.Koch. **(B)** Schematic view. **(C)** The minimal leaf series of sympodial elements in *Philodendron*. **(D)** Shoot with enriched leaf series, including several cataphylls and foliage leaves in *Philodendron*. **(E)** Shoot system in *Dieffenbachia*. Phantom leaves colored black; shoot fusion shown as parallel streaks. Cat, cataphyll; F, foliage leaf; Ifl Ru, inflorescence rudiment; Int, internode, which undergoes intercalary growth under shade avoidance syndrome; LB, lateral bud; Pc Ru, paracladium rudiment in foliage leaf axil (occasional); Sp Ru, spathe rudiment; Sq1, one of two symmetric squamules at base of sympodium element; Sq2, squamule at base of lateral bud; Sym1, Sym2, sequential sympodium elements.

**FIGURE 7:**
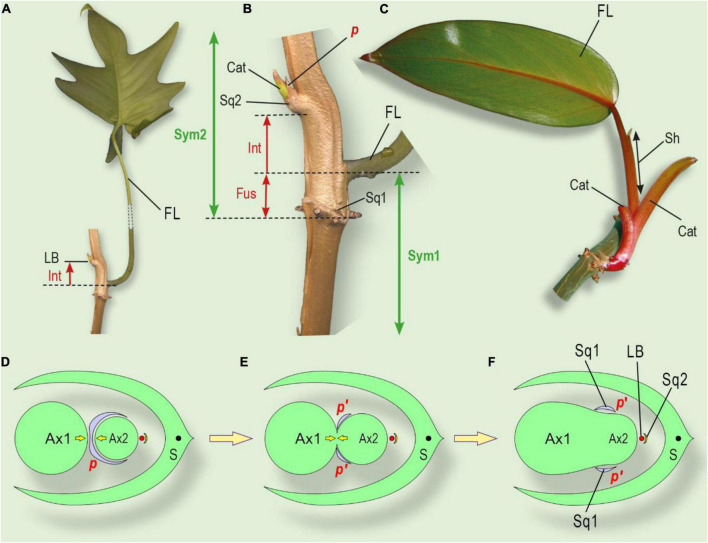
The position of the lateral bud and leaf series of a shoot, grown from the lateral bud and interpretation of squamule formation. **(A)** General view of a shoot system of *Philodendron laciniatum*, exhibiting the shade avoidance syndrome. **(B)** Close-up of lateral bud and sheathing foliage leaf base. **(C)** *Philodendron erubescens*, monopodially growing lateral shoot. Cataphylls gradually increase their size, followed by the foliage leaf. **(D)** The initial state of an integral prophyll under the pressure of two shoots (similar to Figures 5I–L). **(E)** Partial fusion of two axes leads to prophyll splitting (see Figure 5G). **(F)** Complete fusion of two axes with two separate symmetric parts of prophyll (squamulae 1, see Figure 6B). Ax1, parent shoot of I order; Ax2, axillary shoot of II order; LB, lateral bud of III order; *p*, prophyll; S, subtending leaf, corresponding to cataphyll (Figures 6A,B); Sq1 two symmetrically placed squamules at the base of Ax2 (corresponding to splitted prophyll); Sq2, the subtending leaf of the lateral bud. Cat, cataphyll of the lateral shoot; FL, foliage leaf of foregoing element of sympodium; Fus, fusion zone of two sequential sympodium elements; Int, shifting up of the lateral bud due to intercalary growth; LB, lateral bud; *p*, prophyll of lateral bud; *p’*, two separate lobes of the divided prophyll; Sh, a sheath of the foliage leaf; Sq1, two symmetrically placed squamules at the base of sympodial element 2; Sq2, squamule at base of lateral bud (Figure 6B); Sym 1, Sym 2 – sequential elements of sympodium.

The structure of the *Philodendron* shoot system has been attractive to morphologists since the nineteenth century (Irmisch, 1874; Engler, 1877; Engler and Krause, 1912). The remarkable feature of *Philodendron* is the iterative sympodial growth (late in development) with a highly conservative leaf series in each element (Figures 6A,B). It is now widely accepted that the leaf series of the sympodial element consists of a cataphyll, a foliage leaf, and a spathe. The latter belongs to the inflorescence, which is often aborted (Ray, 1987a,b; Mayo, 1991). Every sympodial element bears three potential branching sites: (i) the axil of the cataphyll develops a new sympodial element, (ii) the axil of the foliage leaf bears a lateral inflorescence (paracladium) in some cases, whereas (iii) the axil of the spathe develops no lateral shoots. Additionally, there is another axillary bud that is activated when *Philodendron* is pruned or falls to a horizontal position (Figure 7C). Formally, this bud has no place to be attached to, because all the listed axils are occupied. The question of the position of the lateral bud within the sympodial element has never been broadly discussed in the literature.

A possible interpretation of this lateral bud position is the assumption that besides a common axillary shoot, *Philodendron* produces an adventive (adventitious) bud in the axil of the cataphyll (Mayo, 1991). The axillary shoot develops as the subsequent sympodium element, whereas the adventive one remains as a small dormant bud, which may develop after pruning of the shoot of the sympodium. In this hypothesis, the cataphyll is a true prophyll and bears the ascendant series of lateral shoots in its axil.

Under this interpretation, we would expect some other cases of adventive bud development in the leaf axils of monopodially growing (flagellar) tillers. However, in all cases, every leaf produces a single axillary bud (shoot) and no adventive bud. The foliage leaf of the sympodial element also fails to bear adventive shoots (paracladia). When several paracladia are observed, they are arranged in the manner of a helicoid cyme (branching occurs in bracteole axils). The cataphyll of the sympodium element appears to be the only exception with an adventive bud, which leads us to doubt this hypothesis.

Another expectation is the enrichment of the ascendant series with two or even more adventive buds, as is common in eudicots with serial shoots (e.g., *Lonicera*). However, this expectation also appears to be invalid; we failed to find two adventive buds in the cataphyll axil. It is worth noting that in general, serial adventive shoots are typical for eudicots (both ascendant or descendant), but not for monocots. Monocots usually produce collateral shoots (which is also not the case for the cataphyll of the sympodium in *Philodendron*).

There is another doubt for the traditional interpretation of the cataphyll of the sympodium as a prophyll. Mayo (1991) reported two modes of internode elongation in a sympodial element in *Philodendron*. In subgenus *Meconostigma*, the internode *above* the cataphyll is elongated, whereas, in the subgenus *Philodendron*, the internode *below* the cataphyll is elongated. If we interpret the cataphyll as a prophyll in this branching system, we should accept, that (i) it has a pronounced hypopodium and (ii) this hypopodium is able to elongate. Despite this, the prophylls of the flagellar (monopodial) shoots or the paracladia are sessile (lacking a hypopodium). Furthermore, there are no observations of hypopodium elongation in all these cases. Again, comparing the cataphyll of the sympodium with all the other prophylls in the same plant, we note the exceptional nature of the cataphyll.

The phantom method could help to resolve these problems of the traditional interpretation of the sympodial element in *Philodendron*. First of all, we could postulate one more subtending leaf (Phantom 1) for the axillary bud. However, this assumption is insufficient because the divergence angle between the cataphyll and the axillary bud is 0°, which does not match the divergence angle of approximately 144–180° that is typical for *Philodendron*, as reported by Engler et al. (1990). In order to correct the leaf position, we need one further leaf (Phantom 2) between Phantom 1 and the cataphyll (Figure 6C). In the formal interpretation of the sympodial element, we now have five phyllomes: Phantom 2 (true prophyll, opens the leaf series), Phantom 1, the cataphyll, the foliage leaf, and the spathe. The adaxial prophyll in this case is represented by Phantom 2.

The morphological nature of the cataphyll is elusive. Due to mechanical pressure, it bears two keels and is often bidentate, so was erroneously referred to as a prophyll. In epiphytic *Philodendron* species, if compared with the longer leaf series in the same species, the cataphyll is considerably larger with a longer internode than the sessile scale-like prophyll of the lateral bud. In the lateral shoot series, the cataphylls gradually increase from a small prophyll to the cataphyll of typical size, followed by the foliage leaves (Figures 6D, 7C). The comparison of argumentation of two competing hypotheses (serial buds and phantom hypothesis) are summarized in Table 2.

**TABLE 2:** Comparison of two hypotheses of sympodial element structure in *Philodendron.*

Argumentation/interpretation	Serial bud hypothesis	Phantom prophyll hypothesis
The cataphyll of a sympodial element	A prophyll	Third leaf in a leaf series, starting with two phantoms
The lateral bud position in a sympodial element	In the axil of the cataphyll	In the axil of a phantom
Modes of enrichment of branching of a sympodial element	Two buds from the cataphyll side	One bud from the cataphyll side, the next bud from the opposite side
Organ position after branching enrichment	The cataphylls of the sequential sympodium elements save the angle of divergence	The cataphylls of the sequential sympodium elements change the angle of divergence (according to phyllotaxis)
Buds in the axils of monopodially growing tillers	The upper leaves in a series occasionally develop two or more serial buds	All the leaves produce a single bud
Hypopodium elongation	Possible (and should be observed) in all lateral shoots	Elongation is arrested in all lateral shoots
Predicted results of molecular studies	All the tissues across the bud of a sympodial element express meristem-specific genes	A cryptic subtending leaf of the bud of a sympodial element, expresses leaf-specific genes in early development

Our phantom interpretation is supported by the observation that the axillary bud is placed at a short distance relative to the cataphyll base, but not directly in its axil. Under insufficient light conditions, the lateral bud may shift up its position. This secondary growth occurs late in development as part of a shade-avoidance syndrome, so it cannot be observed at the time of organ initiation. In *Philodendron laciniatum* Engl. we documented the bud even higher than the base of the foliage leaf of the preceding sympodium element. The shift, in this case, is naturally interpreted as an elongation of the internode between Phantom 1 (subtending leaf) and Phantom 2 (true prophyll), which is common in flagellar tillers; the internode between the prophyll and the subsequent leaf elongates freely (Figures 7A,B).

The cataphyll remains the subtending leaf for the next element of the sympodium. The acrotonic branching in the system is noteworthy: the most vigorous lateral stem (the sympodial element) lies in closest proximity to the inflorescence-paracladial zone, the downward axil is occupied by the (dormant) bud, and the most basal axil of the prophyll (Phantom 2) is inactive. This accords with observations of acrotonic branching in other Araceae (e.g., *Anthurium*, *Dieffenbachia*, *Aglaonema*) (Figure 6E). Moreover, acrotonic branching is characteristic of *Philodendron* shoots, derived from the lateral buds (Engler et al., 1990; Choob, 2010).

The best prerequisite in the phantom search that we found in *Philodendron scandens* C. Koch. et Sello is the unpaired scale (squamule) just below the lateral bud. The squamule represents the most likely candidate for Phantom 1. Similar squamules are present in other *Philodendron* species. Phantom 2 (the prophyll) should occupy the opposite position, and two symmetric groups of squamules are visible. As in Amaryllidaceae, the prophyll can be split into two parts (Figure 6E). The construction of the shoot system has led us to assume that two neighboring elements of the sympodium could be partially fused (Figures 7B,D–F). As a result, the axil of the Phantom 2 (prophyll) could not supply an additional bud (Choob, 2010). However, axillary squamules in Araceae cannot be uniformly interpreted – in some taxa they may not be of a foliar nature.

The phenomenon of lateral fusion in *Philodendron* has been largely neglected by botanists. Mayo (1991), illustrating the growth habits of two subgenera of *Philodendron*, drew a short zone after the «prophyll» (cataphyll in the present study), where it is impossible to separate two subsequent elements of the sympodium. The fusion zone becomes more prominent if the plant exhibits a shade avoidance syndrome (Figures 7A,B). A sympodial element has a basal boundary in the place of a cataphyll attachment because the cataphyll is believed to produce a new sympodial element in its axil. The leaf series of a new element consists of the next cataphyll and a sheathing foliage leaf. Thus, the apex of a sympodial element is placed on the level of foliage leaf attachment (Figure 7B). There is a zone of overlap between two sequential elements, which may be referred to as a *fusion zone*. As a consequence of lateral fusion, the adaxial prophyll can split into two symmetric parts, forming axillary squamules (Figures 7D–F). An alternative hypothesis is that the axillary squamules are of a stipular nature.

In *Ph. selloum* K. Koch., fusion occurs between several sequential sympodial units, forming a thick stem. This fusion creates additional difficulties to produce lateral buds, even in the axil of Sq2 (Figure 6B). Thus *Ph. selloum* has a low potential to restore growth after pruning.

Molecular phylogenetic data have placed *Philodendron* in the large *Zantedeschia* clade (Cusimano et al., 2011). We observed the vegetative axillary buds in *Aglaonema* and *Dieffenbachia* from this clade. In both genera, the prophyll was scale-like, with a ring base, sessile, and the hypopodium was not developed. At the same time, the parent axis and the prophyll belong to different physiological domains because of their difference in longevity: prophylls often dry out early in development, whereas the parent stem remains green. We were unable to observe a prophyll with a hypopodium, even among the prophylls of paracladia, and this aspect seems to be the rule for the *Zantedeschia* clade. Our interpretation of the sympodial element is based on the assumption that prophylls in *Phylodendron* are scale-like and lack a hypopodium in all their shoots (Choob, 2010).

## Discussion

The monocot prophyll is a substantial orchestrator of lateral shoot phyllotaxis. The size and shape of the prophyll depend on the taxon, the position of the lateral bud in the whole plant, and the time and mechanical factors of development. These aspects play an essential role in creating morphospace *sensu* De Craene (2018), who distinguished between pressure influence in early and late development. Pressure in early development leads to shifts in pre-patterning and changes in organ positions and rates. The variability in specific patterns of pressure prior to prophyll initiation could represent one reason for high variability in prophyll position (Figures 1C,D,F). The idea of physico-dynamic fields in morphospace is more complex and flexible than predicted by Hofmeister’s rule, which takes into account only distances between organs (Kirchoff, 2000). In some families (Iridaceae, Amaryllidaceae, Commelinaceae) prophyll position is very conservative, resulting in minimal pressure change during the initial steps. During later development, pressure makes certain marks on organ shape (De Craene, 2018). In the monocot prophyll, keels, lobes, incisions, and dissection all represent relatively common features.

In Commelinaceae, the prophyll is a specialized leaf of the vegetative bud, a cataprophyll, which differs from subsequent leaves. With regard to the prophyll indumentum, we observe differences from that of subsequent leaves of the lateral shoot (Figure 3 and Table 1). This means that the prophyll differs at least in the physiological regulation of trichome development. In *Arabidopsis*, the indumentum is indicative of the identity of the phyllomes: branched trichomes are morphological markers of foliage leaves, whereas sepal trichomes are simple, and phyllomes in mutants are often distinguished by differences in trichome development (Mingkun et al., 2015). In the *Arabidopsis* mutants *leafy cotyledon 1* (*lec1*), cotyledons are unable to deposit storage substances, they are desiccation intolerant, and bear ectopic branched trichomes. The uniqueness of the prophyll indumentum may also be interpreted as a sign of its unique identity in the leaf series. At least in some eudicots (*Antirrhinum*), the transcription factor INCOMPOSITA specifically controls prophyll development (Masiero et al., 2004). This factor restricts bracteole growth, so in *inco* mutants the prophylls are increased in size and flower organotaxis is disturbed. Moreover, in the keel region of the prophyll in *Zea mays*, adaxial/abaxial identity appeared to be regulated separately from other parts of the phyllome (Johnston et al., 2010).

In geophytes, the innovation buds are often initiated with prophylls early in the season, when bulbs utilize storage substances and the turgor of the scales of the previous season decreases. The period of primordium development overlaps with nutrient accumulation, bulb thickening, and pressure increase. According to De Craene (2018), this is late-developmental pressure. The scenario of prophyll development depends on scale set and scale longevity in the bulb. In bulbs of daffodils, we even observed one foliage leaf with two separate laminas on a common sheath as a result of strong pressure (Choob and Kozhevnikova, 2000). The prophyll and leaf division into two separate lobes resembles the process of *dédoublement* that is well-documented for flowers (De Craene, 2018). There is a broad spectrum of shapes and sizes that we demonstrated in Amaryllidaceae, ranging from foliage prophylls (photoprophylls) *via* scaly cataprophylls to complete abortion and ablasty. Even in Commelinaceae (Evans et al., 2000), we listed ten genera, including *Commelina*, with partial or complete reduction of the bracteoles (the prophylls of the lateral flowers). Nonetheless, the general organotaxis is not disturbed even in cases of complete prophyll reduction, so in these cases it may be assumed as a cryptic organ, organizing positional information (Choob, 2010, 2020).

The majority of botanists focus their research on visible prophylls only. As a result, in the cases of prophyll reduction, they apply the term prophyll to the first visible phyllome in the leaf series. For example, D. Müller-Doblies introduced the term «abaxial prophyll» to interpret the position of the first leaf of the major innovation bud in *Galanthus* and *Leucojum* (Müller-Doblies, 1971). If we accept his hypothesis, we would need to explain the change from an adaxial (addorsed) prophyll position, typical for other Amaryllidaceae, to an abaxial one. One could predict some intermediate forms with tangential and transversal prophyll positions, rather than a sudden shift. Such changes in prophyll(s) position can be illustrated in *Ranunculus flammula* L., in which the lateral buds develop a single adaxial two-keeled prophyll with a shortened hypopodium in the basal part of the shoot. In the upper shoot region, the hypopodium is longer, the prophyll occupies a tangential position, and one of the keels is gradually reduced. The uppermost part of the shoot with lateral flowers tends to have two transversal prophylls: the second leaf in the series of the lateral flower axis drifts together with the first one, so they appear at the same node as two opposed prophylls. All these events are accompanied by correlative changes in lateral axis organotaxis, suggesting changes in the positional control of leaf development (Choob, 2002, 2010). The «recruitment» of the next leaf to the prophylls in *Ranunculus* illustrates a possible evolutionary pathway from a single prophyll to a two-prophyll state and vice versa.

Among European Amaryllidaceae, we never observed significant deviation of lateral bud phyllotaxis from the overall distichous plane of the bulbs, indicating that positional control remains undisturbed, despite complete prophyll reduction (Choob and Kozhevnikova, 1999, 2000). The proposal of the «abaxial» prophyll by Müller-Doblies (1971) does not explain these facts.

Another example of «the first visible leaf» is the description of the *Philodendron* sympodium (Engler and Krause, 1912; Ray, 1987a,b; Mayo, 1991), where a well-developed cataphyll was claimed to be a prophyll. Comparison with prophylls in the same plant or in closely related Araceae shows that the prophyll is usually sessile, under-developed, and lacks a distinct hypopodium. In contrast, the cataphyll in the *Philodendron* sympodial element is large and possesses a more or less long basal internode. Furthermore, the assumption of this cataphyll as a prophyll does not explain the existence and the position of the latent lateral bud (usually activated after plant damage). The examples of *Galanthus* and *Philodendron* show that «the first visible leaf» concept can lead to false conclusions, and prophyll observations should first be carefully examined from a theoretical viewpoint.

A good example of cryptic leaves occurs in the eudicot family Brassicaceae, in which morphologists believe that the flowers are axillary but the bracts in most cases have been totally ablasted. Tantalizing efforts to locate these bracts using SEM have proved unsuccessful. Nevertheless, a genetic study by Long and Barton (2000) revealed cryptic bracts – cell groups associated with floral primordia, in which genes typical for leaf development (*ANT*) were expressed whereas the genes of the shoot apical meristem (*STM*) were downregulated. It has now been established that under certain conditions, the cryptic bract can develop on a small scale but its further growth is arrested (Müller-Xing et al., 2015). Unfortunately, molecular methods of cryptic leaf search have not yet been applied to European Amaryllidaceae, but these methods could help us to find the addorsed cryptic prophyll of the main innovation bud. Thus, I disagree with the proposal to abolish the term prophyll on the grounds that it is sometimes indistinguishable from other leaves in a leaf series, and it is «just a leaf» (Tomlinson, 1970). In fact, prophylls can play an important role in the positional control of later organotaxis (Choob, 2010).

Plant morphology is based primarily on direct observations of organs in certain positions, combined with analysis of related taxa to build morphological series. This method can help to resolve a direct problem. Another method is to resolve the reverse problem: based on the positions of the subsequent organs, one could predict the position(s) of cryptic organs – sources of positional signals, leading to the observed organotaxis. The axiomatics appear trivial: (i) the prophyll in monocots is adaxial (or slightly deviating); (ii) every lateral shoot has a subtending leaf and prophyll; (iii) the fractional number of phyllotaxis is 0.5 (or some other figure, observed in the taxon). If a cryptic organ is present in the structure, it is possible to point to some contradiction(s) in these postulates. Theoretically, it is necessary to add phantoms to the interpretation scheme, but at a minimal rate. Reanalysis of the phyllomes of the shoot system would draw special attention to the points predicted by the phantoms.

Due to the phenomenon of prophyll reduction, the phantom method has broad application in monocots. We proposed it for Amaryllidaceae (Choob, 1999; Choob and Kozhevnikova, 2000), Iridaceae (Choob and Kuznetsova, 1999; Choob, 2001), and Araceae (Choob, 2010), and it could undoubtedly be applied to many other cases of leaf reduction in monocots. The postulates of the phantom method appear trivial (as trivial as the cryptic bracts in Brassicaceae). Nevertheless, there is no tradition in morphology to extract the consequences of simple postulates and further verify them by observations. The phantom method is a useful predictive tool to search cryptic organ positions, which can be further analyzed using molecular techniques.

## Data Availability Statement

The original contributions presented in the study are included in the article/supplementary material, further inquiries can be directed to the corresponding author/s.

## Author Contributions

The author confirms being the sole contributor of this work and has approved it for publication.

## Conflict of Interest

The author declares that the research was conducted in the absence of any commercial or financial relationships that could be construed as a potential conflict of interest.

## Publisher’s Note

All claims expressed in this article are solely those of the authors and do not necessarily represent those of their affiliated organizations, or those of the publisher, the editors and the reviewers. Any product that may be evaluated in this article, or claim that may be made by its manufacturer, is not guaranteed or endorsed by the publisher.
